# A short form tool for clinical assessment of caregivers’ reaction

**DOI:** 10.1186/s12904-025-01844-w

**Published:** 2025-07-12

**Authors:** Ellen Karine Grov, Bodil Wilde-Larsson, Bente Ødegård Kjøs, Reidun Hov

**Affiliations:** 1 Faculty of Health Sciences, Department of Nursing and Health Promotion, Oslo Metropolitan University, Oslo, 0130 Norway; 2 Department of Health Science, Discipline of Nursing Science, Karlstad University, Karlstad, Sweden; 3 Department of Nursing, University of Inland Norway, Elverum, Norway; 4 Centre for Development of Institutional and Home Care Services, Inland (Hedmark), Hamar Municipality, Norway

**Keywords:** Caregivers, Assessment tools, Short version, The caregiver reaction assessment, Global quality of life

## Abstract

**Background:**

Caregivers take on several tasks to support the patients during their disease trajectory. The price caregivers pay might have impact on their health, schedule, necessity of support from others, economic situation, and quality of life. Therefore, health care personnel need assessment tools to capture the caregivers’ reaction and situation. Clinical practice is characterized as a busy setting and long assessment tools might be time-consuming to complete.

**Methods:**

This study aims to present a short form from an original (long) version of The Caregiver Reaction Assessment (24 items) and to show how these tools correlate in a repeated measures design with three assessment points. Demographics and clinical variables are analysed by means of descriptive statistics. To assess possible sex differences for the long version of The Caregiver Reaction Assessment, the short version of it, and a single item assessing global quality of life, we performed independent sample t-test and Pearson’s correlation analysis.

**Results:**

Sufficient correlation was shown between the long version (24 items), the short version (6 items), and the single item measuring global quality of life. We present how assessment of the caregivers’ reaction and situation can be managed by means of a stepwise approach with one single item on quality of life as the first step, a short form tool on caregiver reaction as the next, and finally a long version of a reputable tool, The Caregiver Reaction Assessment, as the third step.

**Conclusion:**

In this study we have shown how different tools correlate. We suggest these tools to be used in a three-step approach to assess caregivers’ reaction and situation.

**Supplementary Information:**

The online version contains supplementary material available at 10.1186/s12904-025-01844-w.

## Background

Caregivers play a significant role supporting patients with advanced diseases to keep on staying at home [1–3], to reduce the use of formal resources and delay or avoid patients’ admission into care institutions [4]. This is not only cost-effective for the society, but it is also a way to achieve patients` wishes as several studies have shown that seriously ill patients want to stay at home as long as possible [5–7]. The number of caregiving tasks and the length of the caregiving period vary, but with the patients’ presence of comorbidity, advanced treatment and higher life expectancy, it is likely that the caregiving time will increase [4, 8].

The caregivers, who are also named informal, family or primary caregivers [9–11], relatives [12] and next of kins [13], provide most of the assistance and supervision to fulfil the patient’s basic needs [14]. Often, they are the main providers of care to compensate for the patient’s inability to deal with activities of daily living [15, 16]. For instance, they are providing social and emotional support, personal hygiene, food and drink supply [17], shopping and cleaning [18, 19]. Especially for older people suffering from chronic diseases in the palliative phase, their closest caregivers mostly hold a position as practically and emotionally helpers, often leaving them stuck to the caregiver function [14]. Because caregivers tend to lack professional knowledge and have limited care-related training to perform the support, it is likely that they will experience care-induced burden such as depressive symptoms, social isolation, and anxiety [4, 20, 21].

The emotional, physical and psychological costs of caregiving are well documented [22–25]. Studies have found stress, poor mental health, sleep disruption, fatigue, family and social isolation, and limited social activities among caregivers [26, 27]. The consequences experienced by caregivers, might be an evolving concern for the health care system as long as health professionals lack focus on these “silent resources” [4].

The caregivers’ function and role can be defined as “caregiver burden”, understood as the negative value of caregiving [28–30]. However, the opposite dimension “caregiver well-being” or “caregiver reward” might also be present [31, 32]. Even if studies have found consequences both as burden and well-being (reward) among those who have experienced the caregiver function for a family member, it is an advantage to assess the caregiver situation in order to find the most vulnerable caregivers and offer them preventive support and appropriate information [33]. Due to the diversity of caregiver reactions and the variety of offerings to support them, we need assessment tools for clinical evaluation of the caregivers’ situation. Systematic assessment of caregivers is often lacking. In the clinic, lack of time, awareness of community resources, and concerns about patient autonomy are identified as barriers for systematic assessments of caregivers’ situation [34]. To facilitate caregiver assessment, brief self-administered assessment tools and post-screen discussions with clinical health care personnel are recommended [34]. Psychometric validated instruments for assessment of caregivers’ quality of life exist [35–38]. These tools are developed for clinical practice as well as research. For clinical use, it is preferable to perform assessments with brief tools that are not time consuming. Previously, researchers have documented the value of short form assessment tools. The short form tools are valuable and appropriate when they have been psychometrically tested to make sure that they cover the same content as long versions [39, 40]. However, we need more knowledge about which approaches to use, especially in a clinical home care setting as well as evaluation tools on how their situation develops during the caregiver trajectory.

This study was conducted to examine two tools to assess the caregiver situation: a long Norwegian version and a short Norwegian version of The Caregiver Reaction Assessment (CRA) [41] to find whether these tools are covering the same information. By this empirical study we present demographic and relational variables of the sample of caregivers; the scorings on the long and short version of The Caregiver Reaction Assessment, Norwegian version (CRA-N), and the correlation between the long and short version assessed in three waves: at baseline, after 8–10 days and after eight weeks. In addition, we use a single item assessing global quality of life (GQOL) to gain information about the caregivers’ overall situation. We assume that this single item can serve as a first step in assessment of caregivers’ global quality of life [42]. The purpose of this paper is to (1) examine the concurrent validity of the CRA-N long version with the CRA-N short version and Global QOL, and (2) examine test-retest reliability of these tools.

## Materials and methods

### Design

The study design is an instrument development repeated measures with three assessment points.

### Study context and sample

In Norway, as in other western countries, a political goal is to decrease the number of hospital beds [43, 44]. Consequently, the number of patients in palliative phase cared for in their homes is expected to increase.

As long as the home-dwelling patients in Norway need health care, the general practitioner and the home health care service are responsible units. The sample in this study comprises caregivers of home-dwelling older people (≥ 65) with chronic diseases in a late palliative phase [45] and in need of home care nursing service or a cancer coordinator.

There are 356 municipalities in Norway [46]. The municipalities are divided into small (0–9,999 inhabitants), medium-sized (10,000–29,999 inhabitants), and large (30,000 + inhabitants). In this study, we recruited caregivers from 13 municipalities (small/middle-sized (*n* = 9) and large (*n* = 4)) in the Middle-Eastern part of Norway, aiming these municipalities to represent the country.

### The questionnaire

#### Assessment of demographic and relational variables

A demographic sheet was made particularly for this study and consisted of 13 questions: caregivers’ sex, age, native language, educational level, employment status. Also, clinical and relational variables connected to the patient were included concerning groups of diagnoses, duration of the home care nursing service, caregiving responsibility, practical and supportive duties for the patient, duration of caring duties per week, and availability of respite.

#### Instruments

The Caregiver Reaction Assessment (CRA) [41] is an instrument to assess the caregivers’ reaction, originally developed for systematically assessing the caregivers’ situation when caring for patients with physical or mental diseases. Even though Given et al. (1992) mostly describe the assessment tool referring to the caregivers’ reaction and relate to the perspective of family theory, they also mention the caregivers’ situation in their article. As we interpret the concepts, reaction might be the response to the situation and the context in which the caregivers operate. As such reaction and situation are intertwined, and in this research study we find them inextricably linked and use them likewise. Today, this assessment tool is reported to be the most cited in caregiver research [4]. The instrument consists of 24 items that covers five dimensions of the caregivers’ reaction: self-esteem (7 items), lack of family support (5 items), impact on finances (3 items), impact on daily schedule (5 items), and impact on health (4 items). The perceived impact is rated on a 5-point Likert scale, with the format: 1 = strongly disagree, 2 = disagree, 3 = neither agree nor disagree, 4 = agree, 5 = strongly agree. Each subscale is added to a sum score, which divided with the number of items, reflects the unweighted mean-item score with a range from 1.0 to 5.0. The Norwegian version of the CRA was translated into Norwegian and back-translated following the criteria of Acquadro et al. [47]. The psychometric properties of this version of the Norwegian CRA (CRA-N) are documented in Grov et al. (2006) [48]. The assessment tool is validated and reliability tested in Norwegian context with old caregivers and family members caring for cancer patients in the palliative phase [48, 49] as well as by caregivers of patients with cancer in USA [41, 50], Sweden [51], The Netherlands [52], Spain [53], Indonesia [54], Israel [55], and Japan [56]. This assessment tool has been used in several Norwegian studies, e.g., among caregivers of home-dwelling patients receiving home care nursing and caregivers of patients in palliative phase [49], as well as caregivers for patients with different cancer diagnosis [57, 58].

For the clinical purpose, we created a short version of the CRA-N (six items), in which all dimensions of the original CRA were included. Since the dimension “health” might cover both the physical and the mental part, we divided this dimension likewise. The scoring structure follows the Edmonton Symptom Assessment System (ESAS) with the values 0–10 cf [59]. In addition, we added an item on overall wellbeing [42] to this self-assessment tool (with the same scoring structure as the short form), because this item could be seen as an item evaluation the construct validity of the short version of CRA-N. Based on Spilker’s model of levels of quality of life [42], the concept overall wellbeing and global quality of life relates to each other and Scott et al. (2008) find them corresponding [60]. The wordings of the CRA-N short form are “Caregiver: How do you feel right now?” with six lines representing the dimensions ‘Self-esteem’, ‘Family support or support from others’, ‘Finance’, ‘Impact on schedule’, Impact on physical health’, Impact on mental health, and ‘All things considered, how are you today?’. The ratings are scales with descriptions from ‘Best possible’ to ‘Worst possible’ (0–10). English and Norwegian versions of the short form are presented as supplementary material #1.

#### Procedure and data collection

The procedure for data collection was as follows: The nurse leader (responsible nurse for a team/group) or the cancer coordinator in the municipality sampled the informants by identifying the name of the closest caregiver stated in the patient’s journal and informed this caregiver about the study. The caregiver who gave her/his preliminary consent to participate, gave permission to the nurse to bring information so that one of the researchers (RH) or one research assistant could number the questionnaires and send them to the caregiver by post. The caregivers gave their consent to participate by completing and returning the questionnaires.

After that, each caregiver was given a number (to deidentify them as participants in the study) and the names and addresses were maculated after sending out the envelopes. The participants received an envelope containing written information about the study and three stamped envelopes. These envelopes included questionnaires to be completed “to day”/Time 1 (T1) (Background, CRA-N 24 items, CRA-N short version, single item global quality of life [GQOL]), “after 8–10 days”/Time 2 (T2) (CRA-N 24 items, CRA-N short version, GQOL), and “after eight weeks”/Time 3 (T3) (CRA-N 24 items, CRA-N short version, GQOL). Each questionnaire was given the respective participant’s number and grouped in T1, T2, T3 by RH. Data was collected from January 2015 to January 2018.

A total of 373 were invited to the study. Of them 59 caregivers refused to participate, and 8 envelopes were returned unopened. We know that at least 14 patients died during the assessment period, leaving us with the possibility of 292 answers. However, due to several uncompleted answers, both questionnaires (with no imputation), demographic and clinical variables of the patients as well as aspects of the caregivers, we are left with *n* = 199 at T1, 158 at T2 and 124 at T3.

#### Ethics

The study was carried out in accordance with relevant guidelines and regulations, i.e., reported to the Norwegian Social Science Data Services (NSD/Sikt) (Reg.no. 41668) and evaluated to be beyond the scope of the Regional Committee for Medical and Health Research Ethics in South-East Norway (Reg. no. 552367) corresponding to the Declaration of Helsinki. The head of the health care services in each municipality gave local approval for the study.

The caregivers were informed verbally and in writing about the integrity, voluntariness and confidentiality of the study and assured that personal identification would be impossible in the published results. Informed consent was obtained from all participants when answering the questionnaire. The patients were expected to be alive all the three times (extended over eight weeks) when their caregivers should answer the questionnaires. Ethically, due to the risk that patients might die during this period and to avoid risk of re-identification, no lists of names were established. The completed forms have been stored as recommended by the University of Inland Norway, corresponding to national guidelines.

### Data analysis

Data were analysed using IBM SPSS Statistics, version 27. Characteristics of the patients and caregivers were analysed by means of descriptive statistics (frequencies, percentages, mean, median, range and SD). Potential differences between female and male caregivers and the respective items of the long (24 items) CRA-N and the short version (6 items) of CRA-N were analysed with independent sample *t*-test. To examine the concurrent validity of the CRA-N long version with the CRA-N short version and Global QOL, Pearson’s correlation analysis was performed. For examining test-retest reliability of these tools Cronbach’s α was performed.

## Results

The participants had a mean age of 65 (range 37–94 years). A total of 111 (56%) were female. They reported educational level > 12 years (*n* = 72, representing 36%). A total of 111 (56%) were retired or on sick leave. Most of the patients had cancer (*n* = 102, representing 52%). The duration of the home care assistance (≥ 1 year) was reported by 73 (37%). Of practical duties, the caregivers reported that the patient needed assistance to hospital/physician consultation *n* = 153 (82%), paying bills *n* = 126 (68%), easy homework *n* = 123 (66%), and preparing meals *n* = 105 (57%). Caring duties more than 12 h a week were reported by 49 caregivers (27%). Respite from the municipality was reported by 33 (27%) and 59 (47%) reported respite as insufficient. Please see Table 1.

**Table 1:** Demographic and relational variables of the caregivers

Age (in years)	
-Mean	65
-Median	65
-SD	12.6
-Range	37 – 94
	**n (%)**
Gender	
-Male	86 (44)
-Female	111 (56)
Native language	
-Norwegian	198 (99)
-Other	1 (1)
Educational level	
-High (>12 years)	72 (36)
-Low (≤12 years)	127 (64)
Employment status	
-Working	88 (44)
-Retired/sick leave	111 (56)
Patient diagnosis*	
-Cancer	102 (52)
-COPD	38 (19)
-Neurologic (Parkinson/MS)	21 (11)
-Muscular/skeleton	30 (15)
-Heart diseases	33 (17)
-Dementia	21 (11)
-Other	60 (30)
Home care assistance (duration)	
-< 6 months	71 (36)
-≥ 6 months < 1 year	52 (27)
-≥ 1 year	73 (37)
Caregiver responsibilities	
-Yes	175 (91)
-No	18 (9)
Practical duties*	
-Preparing meals	105 (57)
-Personal hygiene	43 (23)
-Easy housework	123 (66)
-Heavy housework	93 (50)
-Administration of medication	64 (34)
-Paying bills (private economy)	126 (68)
-Assistance to hospital/physician consultations	153 (82)
-Other care tasks	83 (45)
Duration of caring duties pr. week	
-0-3 hours a week	46 (26)
-4-8 hours a week	48 (27)
-9-11 hours a week	36 (20)
-≥ 12 hours a week	49 (27)
Respite from caring duties	
-Yes	86 (49)
-No	92 (51)
Respite from whom*	
-Caregivers	60 (49)
-Friends	4 (3)
-Volunteers	8 (7)
-From the municipality health care service	33 (27)
-Others	17 (14)
Sufficient respite	
-Yes	66 (53)
-No	59 (47)

*Possible to answer more than one alternative/item

The caregiver reaction (measured with the CRA-N) for the whole sample and for female and male caregivers are presented in Table 2. Only two items showed differences between female and male caregivers (items no. 12 and no. 21; ‘Never do enough to repay’ and ‘Financial strain’) where female caregivers reported lower scores, interpreted as a worse situation, than male caregivers. The short version CRA-N containing six items, show significant difference between male and female, where female caregivers reported higher impact on schedule than male caregivers. For the single item of global quality of life (GQOL), no significant sex difference was revealed.

**Table 2:** Overview of caregivers’ self-reported scorings on the long CRA-N 24 items version, the six-items CRA-N short version and the global quality of life single item

	Caregivers of chronically ill patients in the late palliative phase			
*CRA-N items*	Total *N* = 197 (100%) mean (SD)	Female *n* = 111 (56%) mean (SD)	Male *n* = 86 (44%) mean (SD)	*p*-value
1. Privilege to care	3.79 (1.02)	3.70 (1.07)	3.86 (0.93)	0.288
2. Others dump caring	2.31 (1.19)	2.28 (1.19)	2.36 (1.20)	0. 670
3. Finances adequate	3.38 (1.21)	3.30 (1.18)	3.46 (1.26)	0.398
4. Activities centred on care	3.33 (1.21)	3.30 (1.23)	3.34 (1.20)	0. 906
5. Tired all the time	3.22 (1.26)	3.32 (1.24)	3.10 (1.28)	0.238
6. Difficult to get help	2.92 (1.32)	3.00 (1.24)	2.84 (1.44)	0.416
7. Resent having to care	2.03 (1.02)	1.96 (1.02)	2.13 (1.02)	0.283
8. Stop work to care	3.15 (1.22)	3.08 (1.22)	3.24 (1.25)	0.369
9. Want to care	3.27 (1.06)	3.24 (1.11)	3.31 (0.99)	0.648
10. Health has gotten worse	2.71 (1.32)	2.73 (1.31)	2.68 (1.35)	0.767
11. Visit family/friends less	3.69 (1.29)	3.64 (1.27)	3.72 (1.35)	0.684
12. Never do enough to repay	3.21 (1.13)	3.03 (1.09)	3.49 (1.09)	**0.004**
13. Family works together	2.69 (1.23)	2.65 (1.23)	2.75 (1.22)	0.599
14. Eliminate from schedule	3.57 (1.29)	3.51 (1.33)	3.65 (1.25)	0.477
15. Physical strength	3.39 (1.22)	3.28 (1.23)	3.55 (1.20)	0.160
16. Feel abandoned	1.95 (1.03)	1.96 (0.99)	1.97 (1.08)	0.941
17. Caring makes me feel good	3.33 (1.00)	3.24 (1.05)	3.46 (0.93)	0.166
18. Interruptions	3.19 (1.27)	3.17 (1.26)	3.22 (1.28)	0.805
19. Healthy enough to care	3.61 (1.10)	3.51 (1.14)	3.76 (1.02)	0.143
20. Caring is important to me	3.60 (1.00)	3.59 (1.03)	3.62 (0.95)	0.858
21. Financial strain	2.14 (1.02)	1.95 (0.85)	2.42 (1.16)	**0.003**
22. Family left me alone	2.71 (1.27)	2.73 (1.19)	2.72 (1.40)	0.964
23. Enjoy caring	3.30 (0.99)	3.30 (0.99)	3.32 (1.00)	0.877
24. Difficult to pay	2.03 (0.95)	1.92 (0.88)	2.20 (1.02)	0.060
*Short version CRA-N (six items)*				
1. Self-esteem	4.17 (2.24)	4.12 (2.19)	4.24 (2.31)	0.710
2. Family support	4.46 (2.63)	4.69 (2.57)	4.20 (2.72)	0.206
3. Finance	3.59 (2.33)	3.83 (2.24)	3.34 (2.38)	0.152
4. Schedule	5.59 (2.35)	6.01 (2.30)	5.06 (2.34)	**0.006**
5. Physical health	4.55 (2.52)	4.72 (2.69)	4.37 (2.29)	0.332
6. Mental health	3.96 (2.57)	4.08 (2.55)	3.84 (2.58)	0.521
*Global Quality of life (single item)*				
How are you today?	4.38 (2.21)	4.48 (2.16)	4.28 (2.28)	0.553

We calculated test-retest reliability (assessed at baseline, after 8–10 days and after eight weeks) which revealed Cronbach’s α 0.724 for the original CRA-Norwegian version, and Cronbach’s α 0.836 for the CRA-N short form. For each dimension test-retest reliability values for the two versions (original CRA-Norwegian version [i.e. long version], and the CRA-N short form) are presented in Table 3.

**Table 3:** Test-retest reliability for CRA-N long and short versions, comparison for the three assessment times

**CRA-N long version**			
Variable/Time points	T1-T2	T1-T3	T2-T3
Self-esteem	0.85	0.83	0.88
Support	0.82	0.80	0.87
Finance	0.70	0.61	0.80
Schedule	0.87	0.79	0.87
Health	0.87	0.78	0.86
**CRA-N short version**			
Variable/Time points	T1-T2	T1-T3	T2-T3
Self-esteem	0.59	0.62	0.68
Support	0.70	0.58	0.56
Finance	0.71	0.64	0.73
Schedule	0.67	0.59	0.69
Health physical	0.78	0.76	0.77
Health mental	0.73	0.67	0.72

Values ≥ 0.70 show high internal consistency which in our study indicate satisfactory internal consistency. In this study we computed convergent validity between the forms (dimensions and GQOL) with correlation analysis. Convergent validity refers to how well a test measures the same or similar constructs. Most of the correlations of the items within the scale are within the range 0.30 − 0.70 which indicate good convergent validity, while for the dimension self-esteem in the CRA long version the values are too low to conclude that the Global Quality of Life single item, has the similar construct. Please see Table 4.

**Table 4:** Correlations: CRA-N long and CRA-N short with GQOL

**Variable/Time points**	**T1**	**T2**	**T3**
**CRA-N long**			
Self-esteem	0.16	0.19	0.15
Support	0.32	0.48	0.44
Finance	0.33	0.35	0.45
Schedule	0.52	0.64	0.63
Health	0.57	0.63	0.66
**Variable/Time points**	**T1**	**T2**	**T3**
**CRA-N short**			
Self-esteem	0.68	0.75	0.78
Support	0.41	0.49	0.51
Finance	0.47	0.53	0.53
Schedule	0.33	0.50	0.49
Health physical	0.59	0.60	0.71
Health mental	0.79	0.78	0.87

The correlation matrix is presented in the supplementary material 2 and shows that the items overlap with values that confirm that the short version of CRA-N cover the same content as the long CRA-N [61]. The exact p-values are given in the matrix to confirm our interpretation. Except for CRA-N dimension 1 & CRA-N short form for assessment 1, and CRA-N dimension 1 & GQOL the correlation matrix shows sufficient correlations.

**Fig. 1:**
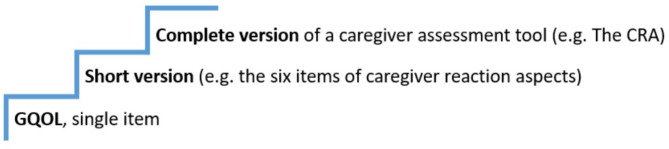
The caregiver assessment steps; advancement of details from a single item, via a short version to a complete (long) assessment tool. CRA = The Caregiver Reaction Assessment, GQOL = Global Quality of Life

Since the correlations between long, short and the single item GQOL indicate overlap between items, we suggest a simple model of assessment approach that we assume is usable in clinical practice. The first step is assessing the GQOL single item. In case of high scores in this first step, which indicate challenges or problems, we recommend going to the next step. In a Norwegian context, when high scores on the second step (CRA-N short version), we turn to step three (CRA-N long version) in order to gain necessary and detailed information from caregivers. Please see Fig. 1 where we suggest the stepwise approach in assessing the caregiver’s situation.

## Discussion

This study was conducted to examine tools to assess the caregivers’ situation: a long version and a short version of The CRA-N [41], and a single item covering global quality of life (GQOL), to find whether these tools are covering the same information. We assume that this single item can serve as a first step in assessment of caregivers’ global quality of life.

By this empirical study we present demographic and relational variables of the sample of caregivers; the scorings on the long and short version of CRA-N and the correlation between the long and short version in addition to the single item (GQOL) assessed in three waves: at baseline, after 8–10 days and after eight weeks.

The value of systematic assessment of the caregivers’ reactions/situation are shown in several studies [34, 62, 63]. Among many available assessment tools different perspectives are found, e.g. quality of life, caregiver burden, coping, and work load [35, 37]. Additionally there exist instruments for particular patient diagnosis and how caregivers can deal with such challenges (e.g [64, 65]).

However, the home care nursing service has to deal with different patient groups, and in old age comorbidity is present in many of the patients [66, 67] which make diagnose specific instruments insufficient. Thus, instruments which cover broad domains are relevant. As Kew and Osborne [35] suggest, the ability of measure caregiver burden is limited because the tools do not capture factors associated with caregiver situation. Therefore, it is crucial to find instruments that cover aspects of interest in particular studies as well as in clinical practice [37]. However, such instruments might be time consuming to complete. Availability of short versions of assessment tools is important for use in clinical practice. Noguchi et al. (2024) have tested a short version of The Caregiver Reaction Assessment (CRA). Their Japanese 10 items-version of the CRA shows good model fit and construct validity [68]. As far as we know, the latter mentioned version is the only short version of the CRA. In addition to the psychometric testing, the authors examined cutoff values for clinical usability in order to identifying high risk caregivers [68]. Cutoff values are valuable clinically, and with possibility to use the tool to compare different target groups, health personnel might shed light on vulnerable caregivers [49]. As such, cutoff values and caregiver group comparison can serve as evidence-based practice and documentation to allocate resources from the health care service.

In a home situation, the patients need to feel safe, and available caregivers might be one contributing factor [69]. Therefore we need to know the situation of caregivers to reach the goal of home-dwelling as long as possible [17]. In our study more than half of the participants reported low education, they were on sick leave or retired and over 65 years of age. We agree with Horsfall et al. [70] that health care professionals should consider caregivers’ backgrounds when they assess the caregiver situation and caregiver burden.

As shown in our results, also other studies have found that female caregivers show higher levels of caregiver burden than male did [71]. This is not surprising, since many of today’s women work outside the home, while the traditional role of women at home may not have been correspondingly changed. Taking into account that the older population is growing, we need also to follow-up the old caregivers as many of them might be unwell themselves and are unable to take on the role as a caregiver [71].

Cooperation with the caregivers’ home care nurses is important [17, 43]. By assessing the caregivers’ situation, we get to know some aspects that are covered in CRA-N, the short and the long version. By using this assessment tool as a way to get to know the caregiver, we can find their involvement in the patient’s situation [71] and possibilities to participate in decision making [2]. As recommended by Given (2019), health care personnel should assess the caregiver’s reaction and situation already by the first assessment of the patient’s needs. After that, regular assessments should be done to detect changes and to follow-up the caregivers’ issues. Supporting caregivers can prevent them from developing health problems and even contribute to improve the quality of patient care.

From this study, we have learned that we can use the three-step approach to assess caregivers’ reaction and situation. Moving from a short single item through a short version to the long tool depends on what the caregiver brings to front. We have shown sufficient correlations between the three steps covering caregivers’ self-reported experience just now. Therefore, we suggest that the three-step approach is valuable in clinical practice where health care personnel have shortage of time doing caregiver assessment. We agree with Kew and Osborne (2023) that suggest interventions and education on aspects of content in assessment tools are warranted.

We have chosen one example with aspects we find relevant for the caregiver situation in a Norwegian context. However, if we want to cover existential or environmental aspects, other instruments are covering these [37].

## Limitations

We found that the sample in our study had high percentage of participants with low education level. In addition, 56% reported being on sick leave or retired. Both findings might explain that some of the participants had problems with completing the questionnaire. Additionally, the caregivers reported that they helped the patient with practical duties as assistance to hospitals/general practitioners (82%), paying bills (68%), easy homework (66%), and preparing meals (57%), which indicate that the patient needs much help from the caregiver. The demographic data shows a large range of age in this study (37–94) which also can serve as a limitation to answer the questionnaires in a follow-up study with three assessment points. The results of this study might have given even worse values because we have no information of the non-responders. We assume that those refusing to participate are in a condition that might be worse than those attending this study.

## Further research

The single item assessing global quality of life, the short version tool CRA-N developed for clinical use to assess caregiver reaction and situation, and the long version CRA-N show sufficient correlation. We assume that the content in these tools cover partly the same; however, to find if global quality of life covers reaction and situation and CRA covers aspects of global quality of life, is left for further examination. A complete overview and synthesis of psychometric properties for CRA used in different countries all over the world could be an interesting next research step since health personnel need solid tools to assess the caregivers’ reaction and situation the coming decades due to the expected increase of old people needing help and support. Insight from other countries will increase this assessment tool’s value. A further suggestion is to establish valid cutoff values to enhance the clinical value of the CRA.

## Conclusions

The short version CRA-N developed for clinical use to assess caregiver reaction and situation shows sufficient correlation with the long version of CRA-N. We also found sufficient correlation between the two latter mentioned tools and a single item on global quality of life. Additionally, we suggest these tools to be used in an approach consisting of three steps: 1. the single item to get a quick overview of the caregivers’ situation, 2. a short version for more detailed information on the caregivers’ reaction and situation, and step three, 3. a long assessment of the caregivers’ reaction and situation.

## Electronic supplementary material

Below is the link to the electronic supplementary material.

Supplementary Material 1

Supplementary Material 2

## Data Availability

No datasets were generated or stored after completing the current study.

## Abbreviations

GQOL: Global Quality of Life
ESAS: The Edmonton Symptom Assessment Scale
CRA: The Caregiver Reaction Assessment
CRA-N: The Caregiver Reaction Assessment – Norwegian version
