# Supplementary material for: A short form tool for clinical assessment of caregivers’ reaction
Source: BMC Palliat Care. 2025 Jul 12;24:198. doi: 10.1186/s12904-025-01844-w (PMC12255985; doi:10.1186/s12904-025-01844-w)
Supplement: Supplementary file 1 — Supplementary Material 1 [file 12904_2025_1844_MOESM1_ESM.docx]

**Supplementary material #1.**

**The Caregiver Reaction Assessment Short form (English and Norwegian versions).**

**Name: Date:**

CAREGIVER

How do you feel right now?

Self-esteem

**Best possible** 0 1 2 3 4 5 6 7 8 9 10 **Worst possible**

Family support or support from others

**Best possible** 0 1 2 3 4 5 6 7 8 9 10 **Worst possible**

Finance

**Best possible** 0 1 2 3 4 5 6 7 8 9 10 **Worst possible**

Impact on schedule

**Best possible** 0 1 2 3 4 5 6 7 8 9 10 **Worst possible**

Impact on physical health

**Best possible** 0 1 2 3 4 5 6 7 8 9 10 **Worst possible**

Impact on mental health

**Best possible** 0 1 2 3 4 5 6 7 8 9 10 **Worst possible**

All things considered, how are you today?

**Best possible** 0 1 2 3 4 5 6 7 8 9 10 **Worst possible**

**Navn: Dato:**

PÅRØRENDE

HVORDAN HAR DU DET NÅ?

Selvfølelse

**Svært bra** 0 1 2 3 4 5 6 7 8 9 10 **Verst tenkelig**

Oppfølging fra familien eller andre nære

**Svært bra** 0 1 2 3 4 5 6 7 8 9 10 **Verst tenkelig**

Økonomi

**Svært bra** 0 1 2 3 4 5 6 7 8 9 10 **Verst tenkelig**

Travelhet i hverdagen

**Svært bra** 0 1 2 3 4 5 6 7 8 9 10 **Verst tenkelig**

Egen fysisk helse

**Svært bra** 0 1 2 3 4 5 6 7 8 9 10 **Verst tenkelig**

Egen psykisk helse

**Svært bra** 0 1 2 3 4 5 6 7 8 9 10 **Verst tenkelig**

Alt tatt i betraktning, hvordan har du det i dag?

**Svært bra** 0 1 2 3 4 5 6 7 8 9 10 **Verst tenkelig**
