# Supplementary material for: A short form tool for clinical assessment of caregivers’ reaction
Source: BMC Palliat Care. 2025 Jul 12;24:198. doi: 10.1186/s12904-025-01844-w (PMC12255985; doi:10.1186/s12904-025-01844-w)
Supplement: Supplementary file 2 — Supplementary Material 2 [file 12904_2025_1844_MOESM2_ESM.docx]

**Supplementary material #2.**

**Correlation matrix of the dimensions of the Long CRA assessment tool, the six-items Short version and the Global Quality of Life single-item for the three assessment times**

|  | **Self-esteem 1** | **Self-esteem S1** | **Self-esteem 2** | **Self-esteem S2** | **Self-esteem 3** | **Self-esteem S3** | **GQOL 1** | **GQOL 2** | **GQOL 3** |
| --- | --- | --- | --- | --- | --- | --- | --- | --- | --- |
| **Self-esteem 1** | 1 | .261  p=.001, n=154 | .846  p<.001, n=111 | .181  p=.042, n=127 | .825  p<.001, n=89 | .242  p=.016, n=99 | .158  p=.051, n=152 | .258  p=.003, n=127 | .215  p=.032, n=99 |
| **Self-esteem S1** |  | 1 | .270  p=.003, n=123 | .589  p<.001, n=156 | .266  p=.008, n=99 | .621  p<.001, n=121 | .683  p<.001, n=193 | .558  p<.001, n=155 | .578  p<.001, n=121 |
| **Self-esteem 2** |  |  | 1 | .170  p=.057, n=125 | .879  p<.001, n=89 | .186  p=.070, n=96 | .118  p=.193, n=123 | .193  p=.029, n=124 | .192  p=.061, n=96 |
| **Self-esteem S2** |  |  |  | 1 | .213  p=.033, n=101 | .679  p<.001, n=122 | .564  p<.001, n=156 | .752  p<.001, n=158 | .649  p<.001, n=122 |
| **Self-esteem 3** |  |  |  |  | 1 | .287  p=.004, n=101 | .096  p=.346, n=99 | .212  p=.034, n=100 | .145  p=.149, n=101 |
| **Self-esteem S3** |  |  |  |  |  | 1 | .566  p<.001, n=121 | .660  p<.001, n=121 | .781  p<.001, n=124 |
| **GQOL 1** |  |  |  |  |  |  | 1 | .715  p<.001, n=155 | .658  p<.001, n=121 |
| **GQOL 2** |  |  |  |  |  |  |  | 1 | .749  p<.001, n=121 |
| **GQOL 3** |  |  |  |  |  |  |  |  | 1 |
|  | **Support 1** | **Support S1** | **Support 2** | **Support S2** | **Support 3** | **Support S3** | **GQOL 1** | **GQOL 2** | **GQOL 3** |
| **Support 1** | 1 | .691  p<.001, n=163 | .823  p<.001, n=128 | .627  p<.001, n=136 | .803  p<.001, n=103 | .565  p<.001, n=108 | .320  p<.001, n=164 | .417  p<.001, n=136 | .443  p<.001, n=108 |
| **Support S1** |  | 1 | .628  p<.001, n=130 | .700  p<.001, n=154 | .603  p<.008, n=105 | .579  p<.001, n=120 | .405  p<.001, n=192 | .379  p<.001, n=154 | .387  p<.001, n=120 |
| **Support 2** |  |  | 1 | .714  p<.001, n=131 | .874  p<.001, n=100 | .679  p<.001, n=104 | .328  p<.001, n=131 | .476  p<.001, n=132 | .450  p<.001, n=104 |
| **Support S2** |  |  |  | 1 | .619  p<.001, n=107 | .555  p<.001, n=121 | .297  p<.001, n=155 | .485  p<.001, n=156 | .421  p<.001, n=121 |
| **Support 3** |  |  |  |  | 1 | .622  p<.001, n=109 | .354  p<.001, n=106 | .449  p<.001, n=107 | .441  p<.001, n=109 |
| **Support S3** |  |  |  |  |  | 1 | .423  p<.001, n=121 | .475  p<.001, n=121 | .510  p<.001, n=124 |
| **GQOL 1** |  |  |  |  |  |  | 1 | .715  p<.001, n=155 | .658  p<.001, n=121 |
| **GQOL 2** |  |  |  |  |  |  |  | 1 | .749  p<.001, n=121 |
| **GQOL 3** |  |  |  |  |  |  |  |  | 1 |
|  | **Finance 1** | **Finance S1** | **Finance 2** | **Finance S2** | **Finance 3** | **Finance S3** | **GQOL 1** | **GQOL 2** | **GQOL 3** |
| **Finance 1** | 1 | .545  p<.001, n=165 | .704  p<.001, n=127 | .561  p<.001, n=137 | .610  p<.001, n=105 | .556  p<.001, n=109 | .328  p<.001, n=164 | .336  p<.001, n=137 | .214  p=.025, n=109 |
| **Finance S1** |  | 1 | .552  p<.001, n=131 | .707  p<.001, n=155 | .462  p<.001, n=108 | .644  p<.001, n=121 | .465  p<.001, n=193 | .365  p<.001, n=155 | .302  p=.001, n=121 |
| **Finance 2** |  |  | 1 | .686  p<.001, n=133 | .800  p<.001, n=102 | .699  p<.001, n=104 | .297  p=.001, n=131 | .354  p<.001, n=132 | .277  p=.004, n=104 |
| **Finance S2** |  |  |  | 1 | .631  p<.001, n=110 | .734  p<.001, n=121 | .470  p<.001, n=155 | .528  p<.001, n=157 | .422  p<.001, n=121 |
| **Finance 3** |  |  |  |  | 1 | .685  p<.001, n=110 | .311  p=.001, n=108 | .381  p<.001, n=109 | .446  p<.001, n=110 |
| **Finance S3** |  |  |  |  |  | 1 | .398  p<.001, n=121 | .460  p<.001, n=121 | .532  p<.001, n=124 |
| **GQOL 1** |  |  |  |  |  |  | 1 | .715  p<.001, n=155 | .658  p<.001, n=121 |
| **GQOL 2** |  |  |  |  |  |  |  | 1 | .749  p<.001, n=121 |
| **GQOL 3** |  |  |  |  |  |  |  |  | 1 |
|  | **Schedule 1** | **Schedule S1** | **Schedule 2** | **Schedule S2** | **Schedule 3** | **Schedule S3** | **GQOL 1** | **GQOL 2** | **GQOL 3** |
| **Schedule 1** | 1 | .256  p=.001, n=160 | .873  p<.001, n=124 | .259  p=.003, n=133 | .789  p<.001, n=98 | .331  p=.001, n=104 | .522  p<.001, n=160 | .565  p<.001, n=132 | .529  p<.001, n=104 |
| **Schedule S1** |  | 1 | .166  p=.059, n=130 | .673  p<.001, n=155 | .215  p=.028, n=104 | .593  p<.001, n=120 | .327  p<.001, n=192 | .358  p<.001, n=154 | .274  p=.002, n=120 |
| **Schedule 2** |  |  | 1 | .313  p<.001, n=133 | .869  p<.001, n=99 | .419  p<.001, n=104 | .543  p<.001, n=131 | .637  p<.001, n=132 | .639  p<.001, n=104 |
| **Schedule S2** |  |  |  | 1 | .302  p=.002, n=107 | .690  p<.001, n=122 | .400  p<.001, n=156 | .497  p<.001, n=158 | .403  p<.001, n=122 |
| **Schedule 3** |  |  |  |  | 1 | .443  p<.001, n=107 | .500  p<.001, n=105 | .520  p<.001, n=106 | .634  p<.001, n=107 |
| **Schedule S3** |  |  |  |  |  | 1 | .391  p<.001, n=121 | .494  p<.001, n=121 | .494  p<.001, n=124 |
| **GQOL 1** |  |  |  |  |  |  | 1 | .715  p<.001, n=155 | .658  p<.001, n=121 |
| **GQOL 2** |  |  |  |  |  |  |  | 1 | .749  p<.001, n=121 |
| **GQOL 3** |  |  |  |  |  |  |  |  | 1 |

|  | **Health 1** | **Physical S1** | **Mental S1** | **Health 2** | **Physical S2** | **Mental S2** | **Health 3** | **Physical S3** | **Mental S3** | **GQOL 1** | **GQOL 2** | **GQOL 3** |
| --- | --- | --- | --- | --- | --- | --- | --- | --- | --- | --- | --- | --- |
| **Health 1** | 1 | .608  p<.001, n=169 | .483  p<.001, n=169 | .866  p<.001, n=126 | .602  p<.001, n=139 | .487  p<.001, n=140 | .780  p<.001, n=104 | .578  p<.001, n=109 | .476  p<.001, n=109 | .574  p<.001, n=168 | .559  p<.001, n=139 | .534  p<.001, n=109 |
| **Physical S1** |  | 1 | .590  p<.001, n=194 | .570  p<.001, n=129 | .776  p<.001, n=155 | .416  p<.001, n=156 | .493  p<001, n=108 | .761  p<.001, n=121 | .443  p<.001, n=121 | .590  p<.001, n=193 | .507  p<.001, n=155 | .450  p<.001, n=121 |
| **Mental**  **S1** |  |  | 1 | .398  p<.001, n=129 | .472  p<.001, n=155 | .734  p<.001, n=156 | .440  p<.001, n=108 | .464  p<.001, n=121 | .665  p<.001, n=121 | .790  p<.001, n=193 | .607  p<.001, n=155 | .517  p<.001, n=121 |
| **Health 2** |  |  |  | 1 | .647  p<.001, n=130 | .498  p<.001, n=131 | .856  p<.001, n=98 | .685  p<.001, n=101 | .551  p<.001, n=101 | .491  p<.001, n=129 | .634  p<.001, n=130 | .641  p<.001, n=101 |
| **Physical S2** |  |  |  |  | 1 | .574  p<.001, n=158 | .573  p<.001, n=110 | .771  p<.001, n=122 | .494  p<.001, n=122 | .464  p<.001, n=155 | .597  p<.001, n=157 | .529  p<.001, n=122 |
| **Mental**  **S2** |  |  |  |  |  | 1 | .517  p<.001, n=110 | .526  p<.001, n=122 | .721  p<.001, n=122 | .588  p<.001, n=156 | .775  p<.001, n=158 | .590  p<.001, n=122 |
| **Health 3** |  |  |  |  |  |  | 1 | .739  p<.001, n=110 | .619  p<.001, n=110 | .516  p<.001, n=108 | .614  p<.001, n=109 | .655  p<.001, n=110 |
| **Physical S3** |  |  |  |  |  |  |  | 1 | .647  p<.001, n=124 | .509  p<.001, n=121 | .686  p<.001, n=121 | .713  p<.001, n=124 |
| **Mental**  **S3** |  |  |  |  |  |  |  |  | 1 | .622  p<.001, n=121 | .715  p<.001, n=121 | .866  p<.001, n=124 |
| **GQOL 1** |  |  |  |  |  |  |  |  |  | 1 | .715  p<.001, n=155 | .658  p<.001, n=121 |
| **GQOL 2** |  |  |  |  |  |  |  |  |  |  | 1 | .749  p<.001, n=121 |
| **GQOL 3** |  |  |  |  |  |  |  |  |  |  |  | 1 |

Explanations: Dimensions/factors of the CRA-N=The Caregiver Reaction Assessment, Norwegian version, named Self-esteem, (Family) Support, Finance, Schedule, Health. S= Short version of each dimension/factor where Health is divided into Physical (health) and Mental (health). GQOL=Global Quality of Life. Numbers 1, 2 and 3 indicate assessment time 1 (baseline), 2 (8-10 days after baseline) and 3 (8 weeks after baseline).
